# The Comparison of the Effects of Sevoflurane Inhalation Anesthesia and Intravenous Propofol Anesthesia on Oxidative Stress in One Lung Ventilation

**DOI:** 10.1155/2014/360936

**Published:** 2014-01-05

**Authors:** Engin Erturk, Selma Topaloglu, Davut Dohman, Dilek Kutanis, Ahmet Beşir, Yucel Demirci, Selcuk Kayir, Ahmet Mentese

**Affiliations:** ^1^Department of Anesthesiology and Intensive Care, Faculty of Medicine, Karadeniz Technical University, 61080 Trabzon, Turkey; ^2^Department of Biochemistry, Faculty of Medicine, Karadeniz Technical University, 61080 Trabzon, Turkey

## Abstract

*Background*. The aim of this study is to compare the effects of sevoflurane and propofol on one lung ventilation (OLV) induced ischemia-reperfusion injury (IRI) by determining the blood gas, ischemia-modified albumin (IMA), and malonyldialdehyde (MDA). *Material and Methods*. Forty-four patients undergoing thoracic surgery with OLV were randomized in two groups (sevoflurane Group S, propofol Group P). Anesthesia was inducted with thiopental and was maintained with 1–2.5% of sevoflurane within the 40/60% of O_2_/N_2_O mixture in Group S. In Group P anesthesia was inducted with propofol and was maintained with infusion of propofol and remifentanil. Hemodynamic records and blood samples were obtained before anesthesia induction (*t*_1_), 1 min before two lung ventilation (*t*_2_), 30 min after two lung ventilation (*t*_3_), and postoperative sixth hours (*t*_4_). *Results*. Heart rate at *t*_2_ and *t*_3_ in Group P was significantly lower than that in Group S. While there were no significant differences in terms of pH and pCO_2_, pO_2_ at *t*_2_ and *t*_3_ in Group S was significantly lower than that in Group P. IMA levels at *t*_4_ in Group S were significantly lower than those in Group P. *Conclusion*. Sevoflurane may offer protection against IRI after OLV in thoracic surgery.

## 1. Introduction

One-lung ventilation (OLV) is usually performed to provide wide surgical area in thoracic surgery. During the OLV hypoxic pulmonary vasoconstriction occurs in nonventilated lung (NVL). While the blood flow of other lobe increases, perfusion and oxygenation of NVL decrease. As a result of this, tissue ischemia occurs in nonventilated site. After resuming two-lung ventilation (2LV), the reperfusion of the blood and reentry of oxygen to ischemic tissue cause sudden and significant increase in reactive oxygen species (ROS) production [1]. Increased ROS induce lipid peroxidation of polyunsaturated fatty acid in biological membranes and plasma lipoproteins [2]. These events, reentry of the oxygen to ischemic tissue and peroxidative reaction of some biological structure, are called ischemia-reperfusion injury (IRI). IRI may cause some cardiac complications [3].

The total antioxidant status (TAS) of human body counteracts oxidative stress. Resuming the 2LV from OLV or after treatment of pneumothorax [4, 5] hydrostatic pressure rises may cause increase in alveolocapillary membrane permeability leading to pulmonary oedema. Bowler et al. reported that TAS was decreased by pulmonary edema fluids in acute lung injuries [6]. It was stated that after 2LV severe oxidative injuries may be important in patients without adequate TAS [1].

There are a lot of studies carried out to prevent IRI [7–14]. Some antioxidant agents can restrain lipid peroxidation and reperfusion injury. Propofol, chemically similar to phenol based free radical scavengers, was used for this purpose [7–11]. On the other hand some studies emphasized that halogenated inhalation agent, sevoflurane, can lead to reduction in IRI [12–14].

After reperfusion of ischemic tissue malonyldialdehyde (MDA), toxic intermediate product of lipid peroxidation and ischemia-modified albumin (IMA) levels increase in blood. Thus both MDA and IMA were used as a marker of IRI studies [2, 9].

The aim of this randomized, prospective, double-blind study is to compare the effects of propofol and sevoflurane on IRI in patients undergoing thoracic surgery in which OLV/2LV was used. MDA, IMA, blood gas levels, and hemodynamics were measured for this purpose.

## 2. Material and Methods

After obtaining the ethics committee approval and patient informed consent the study was carried out in 44 patients, aged between 18 and 65, ASA physical status I or II, undergoing OLV/2LV for thoracic surgery. Sealed envelope method was used for randomization and the patients were divided into two groups (sevoflurane: Group S, *n* = 22 and propofol: Group P, *n* = 22). Patients with ASA score of III or more and severe metabolic, renal, or hepatic diseases, using cigarettes or antioxidant agents, were excluded from the study.

All patients were sedated with 3 mg of midazolam intramuscularly 30 min before the operation. In the operating room, electrocardiography, peripheral arterial oxygen saturation, and invasive arterial blood pressure were monitored. First blood samples for blood gas, MDA, and IMA were obtained and vital parameters were recorded at this time (*t*_1_). Thiopental (6 mg/kg) in Group S and propofol (1.5–2.5 mg/kg) in Group P were used for induction of anesthesia. After the administration of fentanil 2 *μ*/kg and rocuronium 0.6 mg/kg all patients were intubated with double lumen tubes. In Group S anesthesia was maintained with 1–2.5% of sevoflurane within the 40/60% of O_2_/N_2_O mixture. In Group P anesthesia was maintained with total intravenous anesthesia using infusion of 125–250 *μ*/kg/min of propofol and 0.1–0.25 *μ*/kg/min of remifentanil. Ventilation was mechanically controlled and OLV was put into practice for surgical intervention using tidal volume: 6–8 mL/kg, with respiratory rate: 12–20 and fraction of inspired O_2_: 1 adjusted to CO_2_: 35–45 mmHg. After the required procedure was carried out 2LV was resumed from OLV. The blood samples were obtained 1 min before 2LV (*t*_2_) and 30 min after 2LV (*t*_3_). Hemodynamics was also recorded at these intervals. At the end of the operation patients were extubated and transferred to Surgical Intensive Care Unit. Last blood samples and hemodynamic record were obtained at postoperative sixth hour (*t*_4_).

The Kolmogorov-Smirnov test was used to determine normality and homogeneity of data distribution. Parametric data (age, blood pressure, OLV time) were compared using one-way analysis of variation (ANOVA). Nonparametric data were compared using the Kruskal-Wallis test. MDA, IMA, and blood gas analysis were compared using Student's *t*-test between two groups.

## 3. Results

There were no significant differences between the groups with respect to age, sex, and OLV time (Table 1).

Although there were no significant differences between the mean arterial pressures, heart rate at *t*_2_ and *t*_3_ in Group P was significantly lower than the parameters in Group S (*t*_2_; 65.05 ± 11.32, 73.95 ± 13.00, *t*_3_; 62.91 ± 12.21, 72.05 ± 15.57, resp.) (*P* < 0.05) (Table 2).

In blood gas analyses, there were no significant differences in terms of pH and pCO_2_. In Group S, pO_2_ at *t*_2_ and *t*_3_ was significantly lower than Group P (*t*_2_: 151.45 ± 71.85, 240.17 ± 117.43, *t*_3_: 186.55 ± 67.62, 259.51 ± 102.98, resp.) (*P* < 0.01) (Table 2).

**Figure 1:**
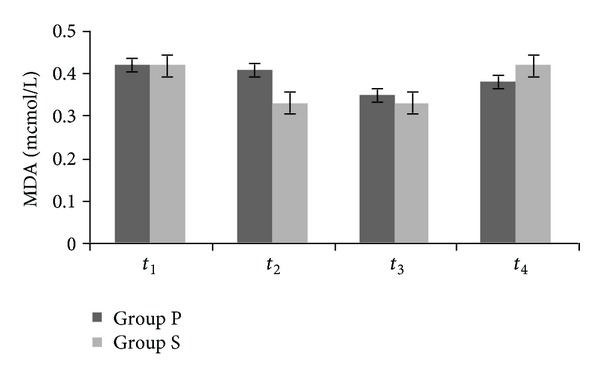
Plasma concentration of malonyldialdehyde (*P* > 0.05).

**Figure 2:**
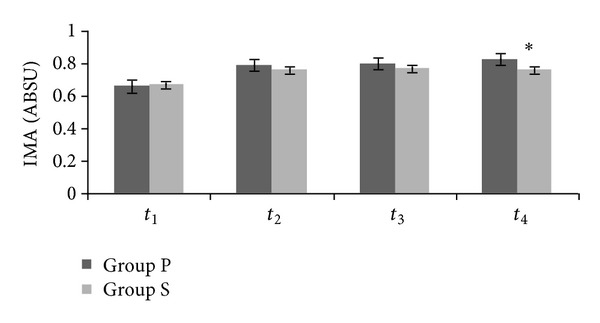
Plasma concentration of ischemia-modified albumin. IMA at *t*_4_ in Group S compared with Group P (*P < 0.05). ABSU: absorbance unit.

There were no significant differences between the groups in terms of MDA (Figure 1). IMA levels at *t*_4_ in Group S were significantly lower than Group P (0.76 ± 0.09, 0.83 ± 0.09, resp., *P* < 0.05) (Figure 2).

## 4. Discussion

This study showed that sevoflurane seemed to provide more protection than propofol by lower increasing in IMA and MDA. These findings have also been clinically important even if there were no complications in patients, because our patients were of ASA I or II score. If this study was performed with ASA III or more scored patients, cardiac or pulmonary complications due to lipid membrane peroxidation could occurr.

Cheng et al. [1] studied the effect of OLV on oxidative stress by measuring ROS and TAS in patients undergoing thoracic surgery with OLV. Their study showed that while ROS increases TAS decreases. In addition authors stated that extravascular lung fluid and intrathoracic blood volume were increased after 2LV. However, mostly patients do not counteract severe complication despite increasing ROS. They explained this condition by the fact that patients with normal TAS can tolerate these negative effects of oxidative stress. However, some critically ill [15], older aged [16], traumatic, or cancer patients have decreased TAS in their plasma. In these patients oxidative stress may cause destruction to DNA and protein and lipid structures.

Propofol was used to decrease IRI in a lot of clinical or experimental reperfusion studies. In an experimental reperfusion model, Akyol et al. [2] found that propofol was effective in protecting lung injury caused by increased oxidative stress and neutrophil accumulation. Huang et al. [8] investigated the effect of propofol infusion anesthesia on reperfusion injury compared to isoflurane inhalational anesthesia during OLV in thoracic surgery. They studied ROS and TAS and stated that propofol infusion shortens and attenuates oxidative stress during OLV. This protective effect of propofol was attributed to its antioxidant properties. However, they also stated that in critically ill patients, the use of total intravenous anesthesia with propofol infusion may be limited because of their unstable hemodynamics. Limited usage of propofol for this reason brings up different agent to prevent IRI.

The effects of inhalational anesthetics on ischemic myocardium have been investigated for many years. The protective effects of halogenated inhalational anesthetics were shown by different studies. Zaugg et al. [17] stated that inhalational anesthetics (sevoflurane and isoflurane) provide protection to IRI in cardiomyocytes by selectively priming K_ATP_ channels through multiple triggering protein kinase C-coupled signaling pathways. In another study, Novalija et al. [18] showed that anesthetic preconditioning with sevoflurane improved adenosine triphosphate synthesis and reduced ROS formation in mitochondria after ischemia by a redox dependent mechanism. One of the good IRI models is cardiac surgery in which reperfusion may cause deterioration of rhythm and contraction of myocardium. Garcia et al. [19] stated at the end of their study that pharmacological preconditioning by sevoflurane provided protective role in cardiac events in coronary bypass patients.

Although there are a lot of studies in different ischemia-reperfusion models, there is no study in which the effect of sevoflurane on IRI was compared with propofol in OLV. Annecke et al. [12] compared the effects of sevoflurane on IRI with propofol after thoracic aortic occlusion in pig. After removing the clamp severe shock occurred in both study groups. While norepinephrine requirements in the sevoflurane group were significantly reduced during reperfusion, serum lactate dehydrogenase, aspartate transaminase, and alanine aminotransferase were also lower with sevoflurane. They state that the use of sevoflurane compared with propofol attenuated the hemodynamic sequelae of reperfusion injury in their model.

Another explanation of protective effect of sevoflurane against IRI may be the effect of it on hypoxic pulmonary vasoconstriction (HPV). During OLV while the perfusion of nonventilated lung is decreased, other lung's is increase. Nonventilated lung remains not only atelectatic, but also hypoperfused and ischemic. While inhalational anesthetics, sevoflurane, can inhibit HPV, intravenous anesthetics, propofol, are unaffected on HPV. Thus, nonventilated lung does not remain severely hypoperfused, and reperfusion injury was limited in patients with sevoflurane anesthesia. Lower pO_2_ levels in Group S at *t*_2_ and *t*_3_ show continuing of the perfusion of nonventilated/atelectatic lung in our patients.

IMA reaches a peak level of sixth hour of reperfusion and begins to decrease at twelfth hour. In this study, lower IMA level in Group S than Group P at postoperative sixth hour showed that sevoflurane provided protection against IRI. However, we did not show the protection with MDA level. There were no different MDA levels between the groups at all measurement times. Cheng et al. [1] stated that resection of lung cancer can decrease MDA levels. Some patients in both groups were operated on for lung cancer. Probably, we cannot support our findings with MDA.

Although these findings encourage us to use sevoflurane to provide protection against IRI, there were limitations in our study. The last blood sample was obtained at postoperative sixth hour. If we investigate postoperative twelfth hour or later, we can show the earlier return to normal IMA level in sevoflurane group. Another limitation of our study is the lacking of another control group. If another control group was formed with an agent with no protection to IRI, the study could be more powerful.

In conclusion we consider that sevoflurane may offer protection against reperfusion injury after one-lung ventilation in thoracic surgery.

## Figures and Tables

**Table 1:** Patients characteristic data.

	Group S	Group P
Age (years)	52.31 ± 13.22	52.45 ± 11.80
Sex (M/F)	6/16	8/14
OLV time (min)	111.59 ± 44.891	135.68 ± 45.021

**Table 2:** Heart rate (HR), mean arterial pressure (MAP), and blood gases.

	*t*_1_	*t*_2_	*t*_3_	*t*_4_
Group S				
HR	81.77 ± 12.78	73.95 ± 13.00	72.05 ± 15.57	79.27 ± 13.93
MAP	90.41 ± 15.00	76.05 ± 9.44	80.64 ± 14.31	84.91 ± 17.84
pH	7.37 ± 0.06	257.31 ± 0.04	7.31 ± 0.03	7.36 ± 0.04
pO_2_	154.95 ± 92.03	151.45 ± 71.85*	186.55 ± 67.62*	147.43 ± 71.41
pCO_2_	44.35 ± 6.75	45.01 ± 8.09	45.15 ± 7.6	39.88 ± 5.99
Group P				
HR	78.41 ± 18.42	65.05 ± 11.32^#^	62.91 ± 12.21^#^	79.45 ± 17.19
MAP	93.05 ± 10.98	81.59 ± 17.37	77.23 ± 16.09	88.32 ± 13.98
pH	7.37 ± 0.04	7.32 ± 0.06	7.33 ± 0.06	7.38 ± 0.03
pO_2_	184.66 ± 83.30	240.17 ± 117.43	259.51 ± 102.98	163.22 ± 64.43
pCO_2_	42.84 ± 6.09	44.38 ± 8.78	42.11 ± 8.09	39.47 ± 4.98

^#^ *P* < 0.05 when heart rate at *t*_2_ and *t*_3_ in Group P was compared with that in Group S.

*P < 0.01 when pO_2_ at *t*_2_ and *t*_3_ in Group S was compared with that in Group P.
